# Associations of cannabis use, other substances, and lifestyle choices on anxiety in medical cannabis patients across 45 days

**DOI:** 10.1038/s41598-026-39086-2

**Published:** 2026-02-26

**Authors:** R. Nathan Pipitone, Benjamin Banai, Jessica Walters, Tyler Dautrich, Kelly Schuller, Martha Rosenthal, Kevin Provost, Branden Hall, Keenan Keeling

**Affiliations:** 1 Department of Psychology, Florida Gulf Coast University, 10501 FGCU Blvd., Fort Myers, FL 33965 USA; 2 Banai Analitika, Josipa Jurja Strossmayera 341, Osijek, 31000 Croatia; 3 CannaMD, 7932 West Sand Lake Road, Suite 205, Orlando, FL 32819 USA; 4 MoreBetter (Releaf App), PO Box 382, Hyattsville, MD 20781-0382 USA; 5 Department of Biology, Florida Gulf Coast University, 10501 FGCU Blvd., Fort Myers, FL 33965 USA

**Keywords:** Anxiety, Daily relief, Real-world observation, Medical cannabis, Lifestyle choices, Other substances, Clinical pharmacology, Psychology, Human behaviour

## Abstract

Previous research has shown medical cannabis (MC) to help reduce anxiety for some individuals, although most studies show trends using cross-sectional designs or conduct secondary analyses of preexisting data. The present study systematically tracked 416 registered MC patients in Florida across 45 days to assess daily shifts in anxiety levels. Using daily recording software, collected data included the frequency of MC use, other substances (e.g., alcohol, anxiolytic medication), other activities (e.g., exercise, meditation), length of previous MC use, and history of anxiety, among other demographic variables. Using linear mixed-effects modeling, results showed that MC use led to the largest anxiety relief across the 45-day period. When participants only used other substances or engaged in activities on certain days, they also saw anxiety reductions, but the major factor in any measurable change in anxiety when comparing different groups to one another was MC use. Age, sex, history of MC use, length of anxiety, and route of MC administration did not significantly contribute to the findings. Sensitivity analyses revealed that novice and experienced MC patients had similar anxiety reductions, although novice patients had slightly better relief outcomes than experienced patients across the 45 days. The anxiolytic effects of MC documented in this study comport with other published work conducting secondary analyses of preexisting data. Although MC can have anxiogenic effects in some individuals, others find it to be an effective way to reduce daily anxiety.

**Supplementary Information:**

The online version contains supplementary material available at 10.1038/s41598-026-39086-2.

## Introduction

Americans feel more anxious with each passing year (with the metric rising from 32% to 37% in 2022 and 2023, respectively, to 43% in 2024)^1^. Of even greater concern, over 31% of U.S. adults will meet the clinical threshold for an anxiety disorder at some point in their lives^2^. Unsurprisingly, first-line treatments, including antidepressants and anxiolytic medications, are facing increased scrutiny. A review of clinical trials by the Food & Drug Administration^3^ supports multiple meta-analyses in the conclusion that antidepressants demonstrate only modest benefits over placebo^4,5^. Likewise, anxiolytic drugs, such as benzodiazepines, have raised red flags for their risk of severe side effects, including psychological and physical dependence^6^.

For this reason, the present study directs its attention to medical cannabis (MC) – an alternative treatment now available under various regulations, in over 42 U.S. states. Previous work suggests cannabinoids (chemical compounds found in cannabis) may dose-dependently induce antidepressant-like effects^7^. Patient behavior appears to align with this theory; in a study of cannabis users, Corroon et al.^8^ found that almost half reported using cannabis as a substitute for prescription medication, with anxiolytics being the second most frequent drug substitution type reported.

Cannabidiol (CBD) and tetrahydrocannabinol (THC) have been the two major cannabinoids examined for their therapeutic potential. CBD has shown to be promising for its anxiolytic effects^9,10^ but see Lichenstein^11^ for mixed results for CBD and THC (the current paper focuses on THC and will use the terms cannabis or medical cannabis (MC) to refer to THC-dominant substances, details in the methods section). Meta-analyses have documented an established relationship between anxiety and cannabis use^12^ suggesting anxiogenic effects and cautions its use for psychiatric disorders^13^, but the causal nature between cannabis and anxiety symptoms remains unclear. When controlling for confounding variables, the relationship does not exist^14^. Even if the relationship does exist, individuals from these studies could have been self-medicating in hopes of reducing their anxiety, which recent work supports^15^. Expectations regarding cannabis’ ability to promote relaxation and reduce tension have also been shown to influence experience, predicting both frequency and severity of use^16,17^. Preliminary PTSD research suggests a psychological component to the effects of cannabis, whereby positive expectancies may contribute to lower levels of anxiety^18^.

Mounting evidence from surveys of medical cannabis patients show that the anxiolytic effects of THC-dominant substances are regularly used and/or reported, whether the product is consumed specifically for anxiety relief or not (e.g., the motivating goal may be to treat pain)^19–21^. Other work shows cannabis to be helpful in relieving stress or assisting in relaxation among medical^22^ and recreational consumers^23,24^ and one study found the initiation and sustained use of MC during a period of three months was associated with decreases in anxiety, compared to controls^25^. Still, other work cautions against using cannabis to treat symptoms of anxiety^26^ – particularly when the product contains high amounts of THC^10^, as anxiogenic effects have been documented^11^.

Considering MC, some limitations of previous work include the retrospective nature in which the data was collected (e.g., memory biases in reflective thinking over weeks and/or months), along with most data being cross-sectional in nature – collected at only one time point^19–21^. More recent work has used smartphone technology to better track real-time (ecological momentary assessment, or EMA) effects that cannabis consumption may have on symptoms like anxiety, with results showing anxiety and stress reduction in most individuals^29–31^. Most participants in these studies used technology to record more than one MC session, thus making it possible to record multiple sessions within individuals across time. With this data structure, more advanced statistical techniques (i.e., panel regression or linear mixed-models) can be used to either control for time-invariant characteristics across multiple individuals who may differ in various ways or simultaneously assess within- and between-participant effects, even when participants provide a different number of observations across time. While some work using these more advanced models has not specifically looked at anxiety changes across time^31^, others have. For instance, Cuttler et al.^29^found no difference in cannabis efficacy (or tolerance effects) across multiple individual sessions. Pipitone at el^30^. factored in MC session amount at the individual level and found that among those who reported anxiety relief, they also reported significantly more sessions and doses per session. While using the more advanced statistical techniques mentioned above allows researchers the ability to calculate average effects within and between individuals, the data was previously collected but analyzed for researcher’s specific purposes), with a wide range of individual responses across time – for example some individuals provide data on more than one occasion (and sometimes on many occasions), while others do not. Systematically tracking individuals over a period of time would provide more within-subject data, thus leading to a better view of how a substance can impact a particular symptom, which is the impetus behind the current study.

## Present study

The goal of the present study is to address previous limitations by tracking changes in Florida medical cannabis patient’s anxiety symptoms over the course of 45 days. Patients used software via their smartphones to track their MC consumption daily, along with any other substances taken or activities engaged in to aid their anxiety. We used Linear Mixed-Effects Modeling (LMM) to analyze the data, which calculates an average effect for each individual based on their aggregate data across 45 days and also across other MC patients within each of the respective groups, described below. We hypothesized that MC would provide overall symptom relief to the majority of patients, similar to previous work^25,29–31^. Because of the study’s longitudinal design, we also wanted to investigate any changes in anxiety across the 45 days.

We also assessed any effect that age and sex would have, particularly for the latter as Cuttler et al.^32^ showed women were more likely to use cannabis to help with symptoms of anxiety and Cuttler et al.^29^ found more anxiety reduction for women than men. However, other work has shown these variables to not play a large role^30^. We were also interested in looking at how alternatives to MC use could influence anxiety changes, as large scale studies on widely utilized activities such as exercise^27^ and meditation^28^ show positive outcomes. Additionally, we were interested in understanding how previous experiences with cannabis would affect anxiety levels, and whether novice MC patients showed any difference from experienced patients. Lastly, we tracked the MC route of administration (ROA), as other work has shown dried flower and higher THC levels to be related to anxiety symptom relief^33^ and others show inhalation of vaporizers or concentrates to be associated with larger increases in emotional well-being^34^.

## Materials and methods

### Procedure

The study was in accordance with the declaration of Helsinki and was reviewed and approved by the Institutional Review Board at Florida Gulf Coast University, protocol #2021-13. Informed consent was obtained from all participants in the study before providing any information. This dataset was provided by MoreBetter. All data came from the state of Florida between July 2022 and December 2022. MoreBetter has been used across the globe by researchers, healthcare professionals, and cannabis product manufacturers to collect real-world data on the use and efficacy of legal cannabis and hemp-derived CBD products. MoreBetter is the creator of the patented Releaf App – a free mobile app that helps individuals monitor their use and experience with cannabinoid-based products, and Penzai – a study management software for remote and decentralized real-world studies. For the present study, we worked with CannaMD, a state-wide network of Florida medical marijuana doctors. To obtain a medical marijuana card in Florida, patients consult with a physician, who determines if they have a qualifying medical condition that meets Florida’s guidelines for medical cannabis use. While Florida law outlines a number of approved conditions for MC treatment (such as cancer and PTSD), legislation also permits MC certification for other conditions of similar like, kind, or class (including anxiety disorders). Patients are only certified under one condition.

Participation opportunities for patients specifically certified under the qualifying condition “anxiety” (meaning patients met their physician’s anxiety disorder diagnostic criteria) were advertised via CannaMD’s newsletter and website, along with in-office reminders from CannaMD staff, who asked patients who qualified for MC as a result of an anxiety diagnosis if they would like to participate. Exclusion criteria for participation included not being 18 years of age or older, not indicating suffering from anxiety, not agreeing to purchase product throughout the duration of the study, and not having access to a smartphone daily. If patients were deemed eligible to participate in the study, they were asked to select a time that worked best for them to answer their daily study questions via SMS messages to their phone. After registering for the study, a message was sent to participants asking them to fill out a questionnaire about previous cannabis and other substance use, the efficacy of those substances in relation to their anxiety, and other demographic characteristics. During the enrollment portion of the study, participants provided information on the type of cannabis product they purchased and consumed on the first day. The majority of participants indicated having THC dominant product compared to CBD levels, and for all participants across all days recorded, most indicated that they used the same product throughout the study, see Table 1 for details.

**Table 1:** Descriptive statistics of variables collected from participants in the study.

Variables	*N*	Percentage or Range	Mean, SD
Total Sample	416		
Age		21–81	41.43, 14.03
18–20	8	2%	
21–30	111	25%	
31–40	120	27%	
41–50	95	21%	
51–60	52	11%	
61–70	53	12%	
71+	8	2%	
Sex			
Male	178	42.8%	
Female	235	56.5%	
Non-binary	3	< 1%	
Previous MC use before study onset	377	91%	5.32*, 8.97*
Patients reporting THC dominant strains	290	86%	
Number of total days using same product	9183	88%	
Drug Group - Participants using other substances	232	52%	
Anti-anxiety medication	110	25%	
Anti-depressant medication	109	24%	
Alcohol	61	14%	
Sleep aid medication	53	12%	
NSAIDS	24	5%	
Muscle relaxers	17	4%	
Narcotic medication	11	2%	
Other substances	87	19%	
Activity Group - Participants engaging in non-substance activities	308	69%	
Exercise	246	55%	
Meditation	176	39%	
Diet	142	32%	
Psychotherapy	70	16%	
Other activity	31	7%	

*in years; MC- Medical Cannabis. Note: Sample sizes and percentages in each of the Drug/Activity Groups include participants in multiple categories, as participants could report consuming (engaging in) different substances (activities) on different days.

After registering for the study, daily SMS messages were sent to participants throughout the 45 days. Individuals were initially asked to select a time in the evening that worked best for them to answer study questions. Based on the time provided, participants received a text message where they could respond to survey questions using Penzai. They were asked about their anxiety before using their MC product (measured on a scale from 0 to 10, with 10 being the most extreme), whether they had used MC or any other substances, and/or engaged in other selected activities (see Table 1). If they had used MC, they recorded the route of administration (ROA). They then provided their anxiety levels after any product used and/or activity engaged in, using the same scale as above. As a form of compensation, participants were entered into a raffle for the chance to receive an amazon gift card at the end of the study. Nine participants were randomly drawn. Two received $250, two received $100, two received $75, and three received $50).

### Participants

A total of 416 participants took part in the study. The average age of participants was 41.43 (*SD* = 14.03) years. During the enrollment portion of the study, participants filled out the Hamilton Anxiety Rating Scale (HAM-A)^35^. The average score across all participants was 29.58 (*SD* = 10.04, *min* = 6, *max* = 56). This score meets the moderate to severe threshold for anxiety (*mild*=below 17, *mild to moderate* = 18–24, *moderate to severe* = 25–30)^36^, thus verifying that participants were dealing with anxiety at the onset of the study. Other descriptive statistics of the variables collected in the study are shown in Table 1, including information on the percentage of participants previously using MC before the onset of the study, percentages and types of other substances used, and percentages and types of activities engaged in.

## Results

All analyses were conducted using Jamovi v.2.5^37^, and its module GAMLj v.3.4.0 for maximum likelihood estimates. Maximum likelihood is a common parameter estimation method in linear mixed-effects models that provides a unified framework for estimating fixed effects, random effects, and variance components simultaneously. Participants were tracked over a period of 45 days on whether they had used MC, other substances, and/or engaged in other activities. The unit of analysis in this study was days, where each participant could record up to 45 days of anxiety changes when using MC, other substances, and/or other activities. To account for repeated measures over the same participants, all statistical estimates were made using linear mixed-effects models with random intercepts specified at the level of the participant.

Altogether, participants logged 11,164 days in the study. Given that 416 participants were tracked across 45 days, this leads to a response rate of 59.64%. To assess whether there were any differences in those who were high or low responders (e.g., non-random missing data) which could have impacted the data, several baseline characteristics were assessed. A median split of 38 days was used to separate patients into low and high respondents. No significant differences were found in age (*t*(414) = 1.66, *p* =.09), current years of MC consumption (*t* (414) = 1.66, *p* =.09) or HAM-A scores (*t*(414) = 1.603, *p* =.110). However, high responders had longer anxiety history (*t*(414) = 2.64, *p* =.009), where high responders had anxiety for 22.3 (*SD* = 14.44) years compared to low responders for 18.41 (*SD* = 15.70) years. There were no differences in novice and experienced MC patients being high and low responders (*χ*^2^ (1) = 0.359, *p* =.549), as well as their sex (*χ*^2^(1) = 3.572, *p* =.059).

First, we inspected the activities that participants logged each day and grouped them accordingly. The largest group included days in which only MC was consumed (*N* = 5502, 49.3%), and this group of days was labeled “Only MC.” The second largest group included days in which participants consumed MC products along with one or more non-drug activities (*N* = 3050, 27.3%) and was labeled “MC + Activity.” Possible activities included: exercise, meditation, diet, psychotherapy, or other activity. The third largest group of days (*N* = 1111, 10%) included those in which participants took one or more medicinal/psychoactive substance (apart from consuming MC) and engaged in one or more non-drugs activities, and was labeled “MC + Drugs + Activity.” Possible substances included: anxiolytic medication, antidepressant medication, sleep aids, narcotics, muscle relaxants, non-steroidal anti-inflammatory substances (NSAIDs), alcohol, and other. The fourth largest group (*N* = 1000, 9%) included days in which participants consumed one or more substances in conjunction with their MC products and was labeled “MC + Drugs.” There was a small proportion of days in which participants recorded responses but did not consume MC products. Among those individuals, 2.2% (*N* = 245) of days included only engaging in one or more activities and no MC, and that group was labeled “Only Activity.” Next, 1.4% of days (*N* = 160) included only consumption of one or more medicinal substances but no MC and was labeled “Only Drugs.” Lastly, in 0.9% (*N* = 96) of days, participants consumed one or more medicinal substance and engaged in one or more activities but no MC, and this group of days was labeled “Drugs + Activity.”

In our first step of analysis, we ran an intercept-only model, with the amount of anxiety relief as a dependent variable. Given the potential differences in nature of events happening during different groups, we ran models separately for each group to estimate average levels of anxiety relief across days within each group, and these results are presented in Table 2. Here, the unstandardized regression coefficients (*b*s), represent the average anxiety relief within each group. Results show that all *b*s were statistically significantly different from 0, which indicates that participants’ anxiety level changed from pre- to post-consumption in all groups in the study, and all were positive which indicated a reduction in anxiety level across days. For the Only MC group, the Cohen’s *d* effect size for mixed effect models^38^ was 1.59, similar to 1.7 found in Stith et al.^31^.

**Table 2:** Intercept only linear mixed effects models for estimating average relief within different groups of anxiety treatment.

Group	*N*_Days_	*N*_Participants_	B	SE	95% CI		df	t	*p*	d	ICC	σ^2^_participants_	σ^2^_residual_
1. Only MC	5502	328	3.56	0.1	3.37	3.75	321.5	35.85	< 0.001	1.59	0.56	2.82	2.19
2. MC + Drugs	1000	159	3.4	0.15	3.1	3.7	122.5	22.37	< 0.001	1.5	0.52	2.68	2.44
3. MC + Activity	3050	260	3.51	0.12	3.27	3.75	245.47	28.66	< 0.001	1.5	0.61	3.32	2.16
4. MC + Drugs + Activity	1111	141	3.02	0.18	2.67	3.38	142.02	16.73	< 0.001	1.25	0.64	3.74	2.08
5. Only Drugs	160	63	1.01	0.21	0.59	1.43	49.08	4.77	< 0.001	0.54	0.48	1.67	1.84
6. Drugs + Activity	96	38	0.95	0.26	0.43	1.47	28.48	3.61	0.001	0.53	0.49	1.58	1.63
7. Only Activity	245	83	1.18	0.14	0.9	1.47	62.19	8.24	< 0.001	0.78	0.45	1.02	1.27

Each row represents a separate intercept-only model for a given group. B- unstandardized regression coefficient; SE- standard error of B; 95% CI- 95% confidence interval; df- degrees of freedom; t- t-test; p- p-value; d- Cohen’s d; ICC- intraclass correlation coefficient; σ^2^_participants_- random intercept variance; σ^2^_residual_- residual variance. Also, Jamovi’s module for linear mixed-effects modeling does not provide standardized regression coefficients as a default output option, therefore only unstandardized betas are provided for all analyses.

Next, we analyzed the whole sample together and entered group type as a fixed effect in the linear mixed effects model, with relief as a dependent variable to assess whether the relief observed among individuals within different groups were significantly different from those who only used MC. The model results are presented in Table 3. The omnibus fixed effect results indicated that significant differences in relief between the groups against the Only MC group existed (*F*(6, 11047.98) = 95.42, *p* <.001). Several patterns can be observed from these results. First, compared to individuals who only used MC, significantly higher relief was seen in groups who used MC and other substances (MC + Drugs) and MC use plus other activities (MC + Activity). Compared to groups who did not use MC (e.g., Drugs Only), groups who used MC showed significantly higher relief, see Table 3.

**Table 3:** Linear mixed effects model for estimating average relief within different groups of anxiety treatment against those using MC only.

Group Comparison	Estimate	SE	95% Confidence Intervals			t	*p*	d
			Lower	Upper	df			
(Intercept)	2.71	0.1	2.52	2.9	540.5	27.82	< 0.001	
2. MC + Drugs − 1. Only MC	0.19	0.07	0.05	0.33	11145.49	2.63	0.008	0.083
3. MC + Activity − 1. Only MC	0.1	0.05	0.01	0.19	11109.52	2.08	0.038	0.043
4. MC + Drugs + Activity − 1. Only MC	0	0.08	−0.16	0.16	11132.55	−0.01	0.994	0.000
5. Only Drugs − 1. Only MC	−1.98	0.14	−2.24	−1.71	10973.4	−14.5	< 0.001	−0.86
6. Drugs + Activity − 1. Only MC	−1.29	0.18	−1.63	−0.94	11031.71	−7.29	< 0.001	−0.56
7. Only Activity − 1. Only MC	−1.97	0.11	−2.19	−1.75	11004.07	−17.53	< 0.001	−0.856
ICC	0.57							
σ^2^_participants_	3.05							
σ^2^_residual_	2.25							

The intercept represents the effect of Only MC entered into the model.

To further assess how each group compared to one another, we proceeded with the interpretation of pairwise comparisons for each group with Bonferroni adjusted *p* values presented in Table 4. There were no significant differences in relief between groups that both consumed MC (e.g., MC Drugs vs. MC + Activity). Also, relief was significantly higher in groups that consumed MC compared to any other group that did not (e.g., Only MC vs. Only Drugs) with the only non-significant group comparisons being when MC was used (e.g., MC + Drugs vs. MC + Activity). Last, among days in which MC was not consumed at all, Drugs + Activity resulted in significantly higher relief compared to Only Drugs and Only Activity days.

**Table 4:** Results of pairwise comparisons of average relief between different anxiety treatment groups.

Group Comparison		d	SE	t	df	*p*_bonferroni_
1. Only MC	2. MC + Drugs	−0.19	0.07	−2.63	11145.49	0.177
1. Only MC	3. MC + Activity	−0.1	0.05	−2.08	11109.52	0.794
1. Only MC	4. MC + Drugs + Activity	0	0.08	0.01	11132.55	1
1. Only MC	5. Only Drugs	1.98	0.14	14.5	10973.4	< 0.001
1. Only MC	6. Drugs + Activity	1.29	0.18	7.29	11031.71	< 0.001
1. Only MC	7. Only Activity	1.97	0.11	17.53	11004.07	< 0.001
2. MC + Drugs	3. MC + Activity	0.09	0.08	1.2	11146.31	1
2. MC + Drugs	4. MC + Drugs + Activity	0.19	0.09	2.23	11096.33	0.545
2. MC + Drugs	5. Only Drugs	2.17	0.14	15.38	10920.01	< 0.001
2. MC + Drugs	6. Drugs + Activity	1.48	0.18	8.17	10,994	< 0.001
2. MC + Drugs	7. Only Activity	2.16	0.13	16.99	11043.12	< 0.001
3. MC + Activity	4. MC + Drugs + Activity	0.1	0.08	1.2	11149.03	1
3. MC + Activity	5. Only Drugs	2.07	0.14	14.96	10976.6	< 0.001
3. MC + Activity	6. Drugs + Activity	1.38	0.18	7.81	11025.14	< 0.001
3. MC + Activity	7. Only Activity	2.07	0.11	18.43	10977.2	< 0.001
4. MC + Drugs + Activity	5. Only Drugs	1.98	0.15	13.47	10995.83	< 0.001
4. MC + Drugs + Activity	6. Drugs + Activity	1.29	0.18	7.18	10990.03	< 0.001
4. MC + Drugs + Activity	7. Only Activity	1.97	0.13	15.22	11091.67	< 0.001
5. Only Drugs	6. Drugs + Activity	−0.69	0.21	−3.31	10934.54	0.02
5. Only Drugs	7. Only Activity	−0.01	0.17	−0.05	10916.63	1
6. Drugs + Activity	7. Only Activity	0.68	0.2	3.5	10965.27	0.01

d- difference; SE- standard error; t- t-value; df- degrees of freedom; p_bonferroni_- Bonferroni adjusted p-value.

Next, we introduced Age and Sex as fixed effects to estimate their contribution to anxiety relief. Prior to this analysis, data for three participants that labeled their sex as non-binary were excluded from the analysis due to small sample size. Results showed that there were no significant effects of Age (*b* = −0.01, 95% CI [−0.02, 0], *p* =.187) or Sex (*b* = 0.22, 95% CI [−0.02, 0], *p* =.187) on anxiety relief for those consuming MC or for any other anxiety treatment group, with full results shown in Supplementary Table 1.

Next, we included Day (ranging from 1 to 45) as a predictor to test whether anxiety relief changed systematically over the study period. Results indicated no significant relationship between Day and relief for the MC group (*b* = 0.0, 95% CI [−0.01, 0], *p* =.068), or for any other treatment group. This suggests that the amount of relief remained stable throughout the 45 days of the study. Full results are presented in Supplemental Table 2.

Next, we entered anxiety history (length of anxiety in years) and length of MC use (in years) as predictors to assess whether these variables contribute to anxiety relief. Anxiety history (*b* = 0.0, 95% CI [−0.01, 0.02], *p* =.488) and length of MC use (*b* = 0.0, 95% CI [−0.03, 0.02], *p* =.859) were not significantly related to changes in anxiety relief. Anxiety history was only a significant predictor for the MC + Activity group, indicating slightly greater relief (*b* = 0.02, 95% CI [0.0, 0.03], *p* =.047) for each year dealing with anxiety. For all other groups, anxiety history was not a significant predictor of overall relief. Full results are presented in Supplemental Table 3.

Next, we entered the route of administration (ROA) as a predictor variable to assess its impact on anxiety relief. ROAs were grouped into five categories: Smoke (60.5% of uses; individuals who inhaled combusted MC with heat using a joint, pipe, or other device), Vape/DAB (23.8% of uses; those who inhaled cannabinoids in more concentrated forms), Smoke and Vape (3% of uses; those who used both of the previous methods simultaneously in one day), Other (11.2% of uses; those who ingested edibles, tinctures, or capsules), and Multiple (1.6% of uses; those who used multiple forms of ingestion simultaneously). Because of sample size limits among the differing categories, we investigated differences between inhalation (Smoke, Vape/DAB, and Smoke and Vape) versus other methods used (e.g., Edibles or Tinctures). Contrasting these two categories of ROAs did not reveal any significant effects on anxiety relief for MC patients (*b* = 0.0, 95% CI [−0.22, 0.23], *p* =.985), nor were there any effects found for the other groups. Full results are presented in Supplemental Table 4. We further explored ROAs by examining another model to see whether there were any differences between those who inhaled regular MC smoke (Smoke, *N* = 6527, 72%) versus those who used concentrates (Vape/DAB, *N* = 2566, 28%). This approach did not reveal any significant differences in anxiety relief for MC patients (*b* = − 0.15, 95% CI [−0.32, 0.03], *p* =.096). Full results can be found in Supplemental Table 5.

In the next model, we wanted to assess whether there were any differences among novice versus experienced MC patients. There were 39 novice patients compared to 401 individuals who had consumed MC prior to the study’s enrollment. There were no significant differences in anxiety relief between novice MC patients and those who had consumed MC previously before the start of the study; full results are presented in Supplemental Table 6. Although overall anxiety relief levels were not significantly different between these two groups, we also conducted a sensitivity analysis of only novice patients over the 45-day period to address any potential prevalent user biases^39,40^. In that cohort, 39 users recorded 936 days of ratings in which 40% were related to Only MC, 45.6% were related to MC + Drugs, MC + Activities, or MC + Drugs + Activities, and the remaining were either Only Drugs, Only Activity, or Drugs + Activity. We entered group as a fixed effect in the model to estimate differences in relief among different groups of novice MC patients. The intercept for the Only MC group (*b* = 2.32) indicated that this group reported significant anxiety relief, see Supplemental Table 7. Furthermore, to explore differences in relief between groups, we looked at post-hoc pairwise comparisons with Bonferroni adjustments. Results showed similar effects to the previous analysis conducted among all patients; relief of groups that combined MC with other Drugs or Activities did not differ from Only MC group, while any group who did not use MC (e.g., Only Drugs) experienced significantly lower relief compared to the Only MC group, see Supplementary Table 8 for full results.

In addition, we explored if novice and experienced MC patients reported similar relief patterns over the 45-day period. We entered participant group (novice or experienced), day of study, and their interaction terms as fixed effects in the model. Participant group by day interaction was statistically significant (*b* = −0.02, *p* =.013). This finding was followed by simple slopes analysis, which is the extraction of the regression line for each group of participants from the existing model. This showed that experienced MC patients had a slight decline in relief ratings (*B* = −0.004, *p* =.016), while novice patients had slight increases in relief ratings (*B* = 0.013, *p* =.052) on average across the study time frame, see Supplemental Table 9 for full results. Last, we assessed the correlation between patient’s history of substance use length and their perceived effectiveness of those substances in dealing with anxiety at the onset of the study. There were significant relationships between substance effectiveness for anxiety and length of use of MC (*r*(399) = 0.15, *p* =.002), antidepressants (*r*(107) = 0.22, *p* =.025), and NSAIDs (*r*(22) = −0.51, *p* =.010). For MC and antidepressants, longer use correlated with better anxiety relief. For NSAIDs, longer use led to less anxiety relief. There were no significant relationships between substance use effectiveness for anxiety and length of use for anti-anxiety medication (*r*(108) = 0.09, *p* =.355), alcohol (*r*(59) = −0.14, *p* =.291), narcotics (*r*(9) = −0.09, *p* =.790), muscle relaxers (*r*(15) = −0.07, *p* =.787), other substances ([e.g., melatonin] *r*(84) = −0.01, *p* =.933), or other prescribed medication (*r*(51) = −0.14, *p* =.319).

## Discussion

**Fig. 1:**
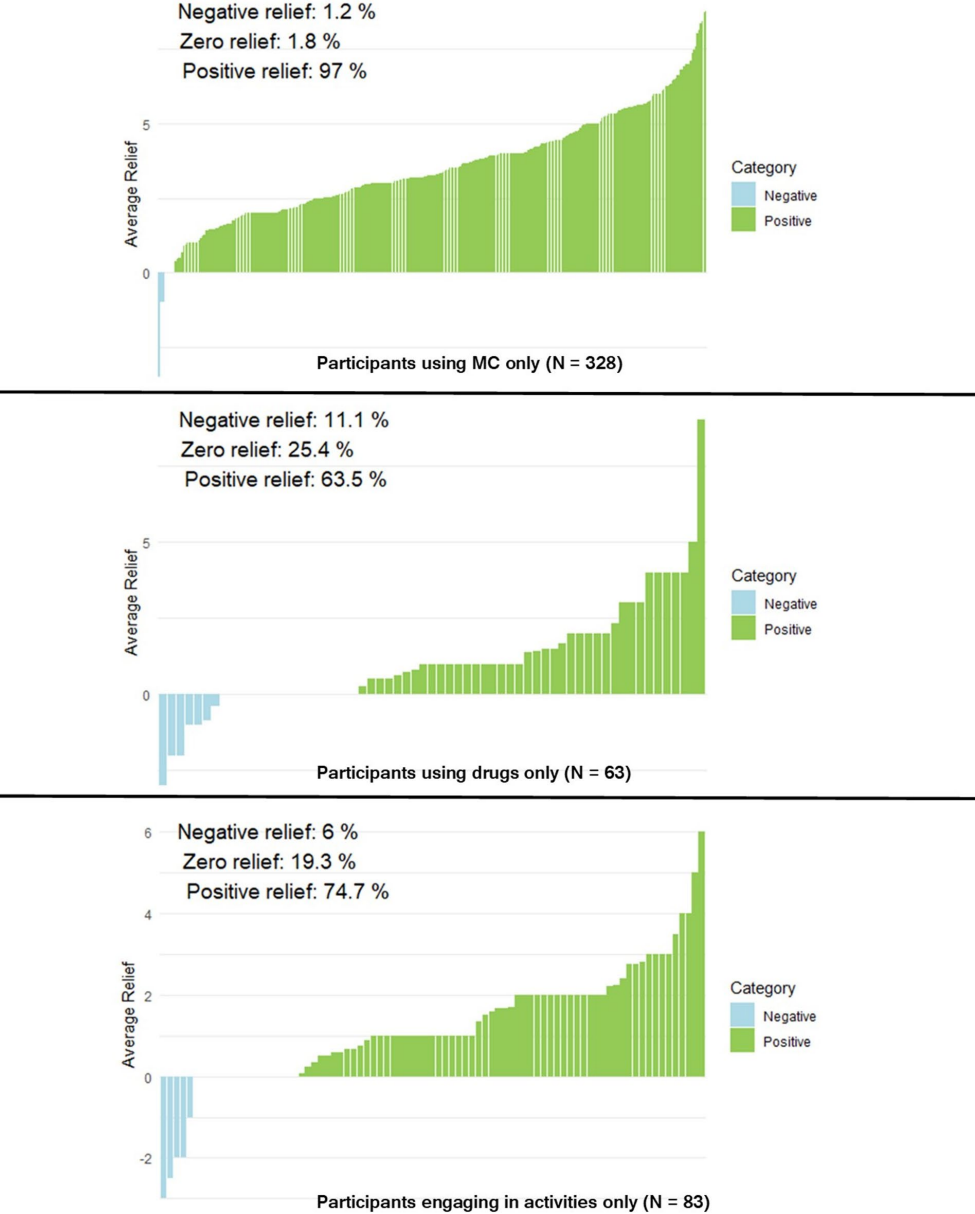
Overall relief experienced across participants reporting using MC only, other drugs only, or engaging in activities only.

The goal of the present study was to investigate daily anxiety changes over 45 days in Florida medical cannabis patients who sought medical cannabis (MC) to relieve their anxiety symptoms. We also tracked other substance use and non-pharmacological activities such as exercise, meditation, and dieting. Using linear mixed effects modeling allowed us to track anxiety changes specific to individuals within each group of use category (e.g., Only MC, MC + Drug) and maximizes sample sizes in each group, as individuals varied in their use patterns and/or activities throughout the 45 days. Results showed that across all 45 days and participants, MC use was the biggest factor in alleviating anxiety. The large effect size calculated from the Only MC group was comparable to other work^31^. Figure 1 shows that almost all participants enrolled in the study found MC to relieve their anxiety. This mirrors findings from Cuttler et al.^29^ who showed that 93.5% of individuals found cannabis to reduce their anxiety, with no changes seen in 4.4% of individuals, and 2.1% of individuals exhibited anxiogenic effects.

Statistically comparing different substance and activity groups against each other showed that the biggest factor in any measurable reduction in anxiety was MC use. When any group without MC use (e.g., Drug Only) was statistically compared to a group using MC (e.g., MC + Drug), the group using MC had significantly higher amounts of anxiety reduction. What is more, no significant differences were found between groups when one group with MC (e.g., MC + Activity) was compared to another group with MC and something else (e.g., MC + Drug). However, this should not insinuate that other substances or engaging in other non-substance activities does not lower anxiety. As shown in Table 2, when analyzed by themselves, participants who used other substances or engaged in other activities alone showed significant reductions in anxiety, see Fig. 1. However, larger anxiety reductions were seen from MC use alone, also shown in Fig. 1. Table 2 shows how MC + Drug and MC + Activity by themselves show significant changes. When they were statistically compared to the Only MC group, they remained significant (Table 3), although these group comparisons did not survive type one error corrections for all pairwise comparisons in Table 4. Also, when participants used other substances and activities in combination on the same day, this led to higher relief compared to those only using substances or activities alone. In other words, there was an additive effect of other substances and activities on anxiety reduction. Other work shows that activities such as exercise^27^ and meditation^28^ by themselves can reduce feelings of anxiety. Based on this evidence and the current study’s findings, alternatives to MC are viable options (by themselves or in combination) for people to consider for reducing their anxiety.

Considering Sex and Age, neither significantly influenced MC’s impact on anxiety for any group studied, which is similar to other findings in previous work^30^. Cuttler et al.^29^ also did not find sex to influence depression symptomatology; however, they did find that women perceived a greater decrease in anxiety symptoms than men. Women persistently have higher prevalence rates of anxiety as compared to men, and the burden of the anxiety is greater^41^. Thus, it would be beneficial for future work to continue to track rates of anxiety relief from MC use as a function of sex.

In looking at how MC impacted anxiety levels across the 45-day period (Days) there was no systematic change across time when entered into the statistical models. This is similar to Cuttler et al.’s^29^ study, which also found no shift across recorded sessions for anxiety (but did find slight increases in depression). Put another way, individuals found MC to be continually effective in reducing their anxiety, with minimal MC tolerance effects observed.

During the enrollment section of the study, participants were also asked to provide their length of time in dealing with anxiety and their length of MC use (if using MC before the onset of the study). Both did not significantly contribute to any change in anxiety relief. Because 39 individuals reported being novice MC patients, we also factored this into our models to assess whether they had different experiences than experienced MC patients. There was no effect of previous use experience with MC on anxiety in general. But we also conducted a sensitivity analysis on the smaller sample of novice MC patients, as prevalent MC patients (experienced patients) are prone to biases in favor of the substance in question (or, patients having negative outcomes with MC are less likely to continue to use the substance and be represented in the data^39,40^. The novice MC patients showed similar findings when comprising different groups (e.g., Only MC, Only Activity) compared to the main analysis with all patients included. But the participation group (experienced vs. novice) and study day interaction was significant, with a simple slopes analysis revealing that novice MC patients had slight increases in their reported relief, compared to experienced MC patients, who showed a slight decrease in their reported relief throughout the study. These effects were smaller and should be interpreted with caution, especially since there were only 39 novice MC patients included in this analysis. Interpreting the beta values from the sensitivity analysis across the 45 days, novice MC patients saw a half-point increase in relief (on a 0–10 scale), whereas experienced users showed approximately a 0.2 reduction in relief. Although these findings are small, they are consistent with other work^25^ which found among anxious individuals, those who initiated MC use over the course of a three-month study showed larger reductions in anxiety compared to those who had sustained MC use before the onset of the study. Other work shows how repeated cannabis use does involve cognitive and physiological tolerance^42^ which could hamper the therapeutic effects of MC over time. Future work looking at longer timeframes, with larger samples of novice MC patients will ultimately help shed more light on these findings and their validity.

Also collected during the enrollment portion of our study was previous use effectiveness of several targeted substances along with the length of use of these substances. Positive correlations were found between length of use of MC effectiveness, and length of use and antidepressant effectiveness. A negative relationship between NSAID use length and effectiveness was also found. The two former results indicate that the longer individuals used MC and antidepressants, the more it seemed to help with their anxiety. For anti-anxiety medication, alcohol, and several other types of medication, length of use did not significantly correlate with effectiveness.

To summarize the immediate findings above, MC continued to lower daily anxiety levels in a similar fashion over 45 days. Although length of previous MC use did not impact daily anxiety relief, individuals who reported using MC longer reported it to be more impactful at the onset of the study even though the sensitivity analysis revealed slight decreases of relief across the study. And, while novice MC patients showed similar anxiety relief reductions compared to those who were experienced, the sensitivity analysis along with other work^25^ has shown it to be more impactful among novice individuals. This could be interpreted as meaning that MC, when used over the long term, may have a reduced ability to improve symptoms like anxiety, or it could simply be that some individuals have high expectations for MC to help with their anxiety initially. Reviews on the effects of cannabis tolerance mainly point towards decreased effects among those who use regularly, although tolerance to the cognitive effects are more prominent compared to the physiological effects^42^. Future work should help paint a clearer picture of how initiating and maintaining MC use helps individuals deal with daily anxiety levels.

Investigating how participants administered their MC revealed no effect of route of administration (ROA) on anxiety relief. Pipitone et al.^30^ found very small effects/no effect of ROA at the symptom/individual level of analysis, respectively. Stith et al.^33^ also found no impact of ROA on general symptom relief. However, in some sense this is surprising, as different ROA coincide with different cannabinoid concentrations entering the system (e.g., regular flower inhalation [Smoke] vs. concentrate inhalation [VAPE/DAB])^43^. As such, Stith et al.^33^ found higher THC levels led to better symptom relief and Cuttler et al.^29^ found higher THC and CBD levels led to more stress reduction. At the same time, MC patients often quickly adjust and refine which products and ROA work better for them^44^. Therefore, the current study may simply have documented how MC provides anxiety relief *when participants use their preferred ROA of choice*. Experimentally manipulating ROA in future work would be an interesting avenue to pursue to understand exactly which ROA may lead to better therapeutic outcomes.

It should be mentioned that there is conflicting data about cannabis’s impact on anxiety. Some studies have found that cannabis (especially cannabis with higher levels of THC) may be anxiogenic^11,12^. If cannabis is used to treat anxiety and depression, the dosage and CBD: THC ratio are important considerations, as are frequency of use, age, and expectations. Due to cannabis’s biphasic dose response curve, low to moderate doses may help alleviate anxiety in some people, but high levels of THC can increase fear, anxiety, and paranoia^10,45^. Frequency of use may play a role as well; anxiogenic effects may attenuate among those who use cannabis regularly^11^. Seniors are one of the fastest growing populations of cannabis consumers in the U.S. Most older adults primarily use cannabis for medical purposes, to treat pain, depression and/or anxiety, and sleep disturbances^46^. Due to age-related changes in the body, as well as the potential for medication interactions, older patients using cannabis may be at greater risk of anxiety from cannabis use. A patient’s expectations may play a role as well. In one study^47^, healthy adults were given sublingual doses of hemp seed oil. In one session, they were told the oil contained CBD, in another session they were told it did not. In reality, neither sample contained CBD. Patients reported less stress when they believed they consumed CBD, and those who most strongly believed that CBD reduced anxiety experienced the lowest levels of anxiety when consuming what they thought was a CBD-containing sample. Studies like this and others^16–18^ show how important expectancy effects are when trying to establish a drug’s true effect on cognition and/or behavior, thus caution is needed when interpreting results from studies that do not use balanced placebo designs/conduct true experiments^48^.

## Limitations

Our results show a consistent trend for MC to alleviate anxiety for a period of 45 days. However, there are limitations to our findings. First, our study design lacked the qualities of a true experiment, namely no random assignment to groups was established, hence no systematic manipulation of MC use or any other drug/activity. One strength in the data collected was the sample (albeit small) of novice MC patients in which a sensitivity analysis was conducted. However, even with this analysis included, we did not have new patient’s expectations of how MC would impact their anxiety, thus we were not able to address the likely confound of expectancy effects. Related to this, we did not consider possible carryover effects of individuals consuming and not consuming MC in close succession, thus ignoring possible pharmacodynamic carryover effects. This is a limitation of nearly all human drug research outside of clinical trials, and until these studies can be conducted, causality will always be less certain.

Second, these participants were recruited from an organization linked to MC doctors (CannaMD), which means most participants were already seeking MC treatment. Thus, we did not have a random sample of individuals and biased expectancy effects towards the therapeutic effectiveness of MC could have impacted the results. However, we did collect data on participants’ previous use of MC and did have a small number of people who reported never using MC previously for their anxiety symptoms with comparable anxiolytic effects seen in this group, although slight differences across the study were seen among novice and experienced MC patients.

We also did not inspect different ratios or amounts of cannabinoids (e.g., THC, CBD) like previous work^29^ which found that high CBD and THC levels facilitated stress reduction (but no linear changes were seen in anxiety). Other recent work has shown higher CBD levels to be slightly more helpful for symptoms of depression and anxiety^9,30^ and higher THC to be associated with better depression and anxiety relief^33^. Systematically altering products with varying cannabinoid ratios across time would help the scientific community understand how different combinations and/or ratios of cannabinoids like THC and CBD ultimately impact symptoms of anxiety.

We also did not differentiate between individual medications and non-pharmacological activities because of sample size issues within individual groups. It would be interesting to assess which specific medications (e.g., anti-anxiety or anti-depressant) and which activities (e.g., exercise or diet) led to the most relief throughout the study, or even what types of exercise regiments or which types of diets (healthy eating or food restrictions) had the biggest impact. Future large-scale work will hopefully be able to partition and investigate these factors further.

### Summary

There is conflicting data about cannabis’s effects on anxiety. Some studies postulate that cannabis (notably higher levels of THC) may lead to anxiogenic effects^10,11,13,49^, although other studies have questioned the causality of the relationship^50^. Some work shows no increase in anxiety when controlling for confounding demographic variables^14^ and still others show it can reduce anxiety symptoms^25^. The present findings show daily anxiety reductions from MC use when tracking individuals across a period of 45 days. This comports with other work^29–31^ analyzing preexisting (EMA) data on same-day pre- and post-cannabis use ingestion via smartphones showing how, at least for some individuals, MC helps to reduce symptoms of anxiety.

## Supplementary Information

Below is the link to the electronic supplementary material.

Supplementary Material 1

## Data Availability

All data will be provided at the request of the reader by contacting the corresponding author.
