# Supplementary material for: Associations of cannabis use, other substances, and lifestyle choices on anxiety in medical cannabis patients across 45 days
Source: Sci Rep. 2026 Feb 26;16:11124. doi: 10.1038/s41598-026-39086-2 (PMC13046955; doi:10.1038/s41598-026-39086-2)
Supplement: Supplementary file 1 — Supplementary Material 1 [file 41598_2026_39086_MOESM1_ESM.docx]

Supplementary Table 1. Linear mixed effects models for estimating average relief within different anxiety treatment groups, with age and gender entered as predictor variables.

| Groups | N_Sessions_ | N_Participants_ | Fixed effects | *b* | SE | 95% CI | | *df* | *t* | *p* | ICC | σ^2^_participants_ | σ^2^_residual_ |
| --- | --- | --- | --- | --- | --- | --- | --- | --- | --- | --- | --- | --- | --- |
| 1. Only MC | 5433 | 325 | (Intercept) | 3.58 | 0.1 | 3.38 | 3.78 | 318.31 | 35.12 | < .001 | 0.56 | 2.82 | 2.18 |
|  |  |  | AGE | -0.01 | 0.01 | -0.02 | 0 | 322.03 | -1.32 | 0.187 |  |  |  |
|  |  |  | Gender (ref. Female) | 0.22 | 0.21 | -0.18 | 0.63 | 317.01 | 1.09 | 0.278 |  |  |  |
| 2. MC + Drugs | 996 | 158 | (Intercept) | 3.41 | 0.16 | 3.1 | 3.72 | 122.09 | 21.35 | < .001 | 0.53 | 2.68 | 2.42 |
|  |  |  | AGE | -0.02 | 0.01 | -0.04 | 0.01 | 119.92 | -1.5 | 0.136 |  |  |  |
|  |  |  | Gender (ref. Female) | 0.07 | 0.32 | -0.57 | 0.7 | 122.61 | 0.21 | 0.835 |  |  |  |
| 3. MC + Activity | 3050 | 260 | (Intercept) | 3.51 | 0.12 | 3.27 | 3.76 | 242.37 | 28.37 | < .001 | 0.61 | 3.32 | 2.16 |
|  |  |  | AGE | -0.01 | 0.01 | -0.02 | 0.01 | 240.2 | -0.79 | 0.431 |  |  |  |
|  |  |  | Gender (ref. Female) | 0.19 | 0.25 | -0.3 | 0.67 | 242.7 | 0.75 | 0.456 |  |  |  |
| 4. MC + Drugs + Activity | 1111 | 141 | (Intercept) | 2.99 | 0.19 | 2.62 | 3.35 | 140.07 | 16.1 | < .001 | 0.64 | 3.76 | 2.08 |
|  |  |  | AGE | -0.01 | 0.01 | -0.03 | 0.02 | 142.67 | -0.7 | 0.485 |  |  |  |
|  |  |  | Gender (ref. Female) | -0.26 | 0.37 | -0.98 | 0.47 | 139.68 | -0.69 | 0.491 |  |  |  |
| 5. Only Drugs | 160 | 63 | (Intercept) | 1.02 | 0.23 | 0.57 | 1.46 | 42.46 | 4.5 | < .001 | 0.48 | 1.72 | 1.84 |
|  |  |  | AGE | 0.01 | 0.01 | -0.02 | 0.04 | 50.27 | 0.72 | 0.472 |  |  |  |
|  |  |  | Gender (ref. Female) | 0.24 | 0.45 | -0.66 | 1.13 | 43.89 | 0.52 | 0.605 |  |  |  |
| 6. Drugs + Activity | 96 | 38 | (Intercept) | 0.94 | 0.26 | 0.41 | 1.46 | 26.65 | 3.53 | 0.002 | 0.5 | 1.62 | 1.63 |
|  |  |  | AGE | -0.02 | 0.02 | -0.06 | 0.02 | 26.8 | -1.03 | 0.313 |  |  |  |
|  |  |  | Gender (ref. Female) | 0.3 | 0.54 | -0.77 | 1.36 | 26.31 | 0.55 | 0.585 |  |  |  |
| 7. Only Activity | 244 | 82 | (Intercept) | 1.18 | 0.14 | 0.9 | 1.46 | 61.03 | 8.29 | < .001 | 0.43 | 0.96 | 1.26 |
|  |  |  | AGE | 0 | 0.01 | -0.02 | 0.02 | 56.95 | 0.04 | 0.966 |  |  |  |
|  |  |  | Gender (ref. Female) | 0.56 | 0.28 | 0 | 1.13 | 59.96 | 1.98 | 0.052 |  |  |  |

B – unstandardized regression coefficient; SE – standard error of B; 95% CI – 95% confidence interval, df- degrees of freedom, t- test, p- p-value; ICC- intraclass correlation coefficient; σ^2^_participants_- random intercept variance; σ^2^_residual_- residual variance

Supplemental Table 2. Linear mixed effects models for estimating average relief within different anxiety treatment groups, with day of study entered as the predictor variable.

| Groups | N_Sessions_ | N_Participants_ | Predictor | *b* | SE | 95% CI | | *df* | *t* | *p* | ICC | σ^2^_participants_ | σ^2^_residual_ |
| --- | --- | --- | --- | --- | --- | --- | --- | --- | --- | --- | --- | --- | --- |
| 1. Only MC | 5502 | 328 | Intercept | 3.55 | 0.1 | 3.35 | 3.75 | 321.96 | 35.66 | < .001 | 0.56 | 2.83 | 2.19 |
|  |  |  | Day | 0 | 0 | -0.01 | 0 | 5330.94 | -1.82 | 0.068 |  |  |  |
| 2. MC + Drugs | 1000 | 159 | Intercept | 3.4 | 0.15 | 3.1 | 3.7 | 122.91 | 22.32 | < .001 | 0.52 | 2.68 | 2.44 |
|  |  |  | Day | 0 | 0 | -0.01 | 0.01 | 917.71 | -0.3 | 0.762 |  |  |  |
| 3. MC + Activity | 3050 | 260 | Intercept | 3.5 | 0.12 | 3.26 | 3.74 | 246.54 | 28.59 | < .001 | 0.61 | 3.32 | 2.16 |
|  |  |  | Day | 0 | 0 | -0.01 | 0 | 2906.92 | -0.26 | 0.794 |  |  |  |
| 4. MC + Drugs + Activity | 1111 | 141 | Intercept | 3 | 0.18 | 2.65 | 3.36 | 142.41 | 16.62 | < .001 | 0.64 | 3.73 | 2.08 |
|  |  |  | Day | -0.01 | 0 | -0.01 | 0 | 1043.79 | -1.71 | 0.087 |  |  |  |
| 5. Only Drugs | 160 | 63 | Intercept | 1.01 | 0.21 | 0.59 | 1.42 | 47.61 | 4.81 | < .001 | 0.46 | 1.61 | 1.86 |
|  |  |  | Day | 0.01 | 0.01 | -0.01 | 0.04 | 155.48 | 1.18 | 0.239 |  |  |  |
| 6. Drugs + Activity | 96 | 38 | Intercept | 0.95 | 0.26 | 0.42 | 1.47 | 28.11 | 3.58 | 0.001 | 0.49 | 1.6 | 1.65 |
|  |  |  | Day | 0 | 0.01 | -0.03 | 0.03 | 93.98 | -0.04 | 0.972 |  |  |  |
| 7. Only activity | 245 | 83 | Intercept | 1.18 | 0.15 | 0.89 | 1.46 | 62.54 | 8.09 | < .001 | 0.45 | 1.04 | 1.27 |
|  |  |  | Day | 0 | 0.01 | -0.02 | 0.01 | 242.15 | -0.5 | 0.615 |  |  |  |

B – unstandardized regression coefficient; SE – standard error of B; 95% CI – 95% confidence interval, df- degrees of freedom, t- test, p- p-value; ICC- intraclass correlation coefficient; σ^2^_participants_- random intercept variance; σ^2^_residual_- residual variance

Supplemental Table 3. Linear mixed effects models for estimating average relief within different groups of anxiety treatment, with anxiety history and MC use length entered as predictor variables.

| Groups | N_Sessions_ | N_Participants_ | Predictor | *b* | SE | 95% CI | | *df* | *t* | *p* | ICC | σ^2^_participants_ | σ^2^_residual_ |
| --- | --- | --- | --- | --- | --- | --- | --- | --- | --- | --- | --- | --- | --- |
| 1. Only MC | 5502 | 328 | (Intercept) | 3.57 | 0.1 | 3.37 | 3.76 | 316.06 | 35.52 | < .001 | 0.56 | 2.84 | 2.19 |
|  |  |  | Anxiety History | 0 | 0.01 | -0.01 | 0.02 | 321.59 | 0.69 | 0.488 |  |  |  |
|  |  |  | MC Use | 0 | 0.01 | -0.03 | 0.02 | 315.9 | -0.18 | 0.859 |  |  |  |
| 2. MC + Drugs | 1000 | 159 | (Intercept) | 3.4 | 0.15 | 3.1 | 3.7 | 120.06 | 22.29 | < .001 | 0.52 | 2.69 | 2.44 |
|  |  |  | Anxiety History | 0.01 | 0.01 | -0.01 | 0.03 | 129.54 | 0.57 | 0.57 |  |  |  |
|  |  |  | MC Use | -0.03 | 0.02 | -0.07 | 0.01 | 101.6 | -1.4 | 0.165 |  |  |  |
| 3. MC + Activity | 3050 | 260 | (Intercept) | 3.51 | 0.12 | 3.27 | 3.75 | 243.27 | 28.54 | < .001 | 0.6 | 3.3 | 2.16 |
|  |  |  | Anxiety History | 0.02 | 0.01 | 0 | 0.03 | 242.81 | 1.99 | 0.047 |  |  |  |
|  |  |  | MC Use | 0 | 0.02 | -0.04 | 0.03 | 231.17 | -0.24 | 0.814 |  |  |  |
| 4. MC + Drugs + Activity | 1111 | 141 | (Intercept) | 3.01 | 0.18 | 2.65 | 3.37 | 136.93 | 16.49 | < .001 | 0.64 | 3.77 | 2.08 |
|  |  |  | Anxiety History | 0.01 | 0.01 | -0.01 | 0.03 | 148.09 | 0.81 | 0.418 |  |  |  |
|  |  |  | MC Use | -0.01 | 0.02 | -0.06 | 0.03 | 118.96 | -0.57 | 0.568 |  |  |  |
| 5. Only Drugs | 160 | 63 | (Intercept) | 1.03 | 0.22 | 0.6 | 1.46 | 46.67 | 4.72 | < .001 | 0.49 | 1.75 | 1.84 |
|  |  |  | Anxiety History | -0.01 | 0.02 | -0.04 | 0.02 | 50.98 | -0.46 | 0.65 |  |  |  |
|  |  |  | MC Use | 0 | 0.03 | -0.05 | 0.05 | 63.01 | -0.11 | 0.909 |  |  |  |
| 6. Drugs + Activity | 96 | 38 | (Intercept) | 0.95 | 0.27 | 0.42 | 1.49 | 27.42 | 3.54 | 0.001 | 0.51 | 1.71 | 1.62 |
|  |  |  | Anxiety History | 0.01 | 0.02 | -0.03 | 0.06 | 29.45 | 0.61 | 0.547 |  |  |  |
|  |  |  | MC Use | -0.03 | 0.04 | -0.1 | 0.05 | 28.91 | -0.69 | 0.495 |  |  |  |
| 7. Only activity | 245 | 83 | (Intercept) | 1.16 | 0.15 | 0.87 | 1.45 | 62.63 | 7.86 | < .001 | 0.46 | 1.06 | 1.26 |
|  |  |  | Anxiety History | 0 | 0.01 | -0.02 | 0.02 | 60.48 | 0.04 | 0.965 |  |  |  |
|  |  |  | MC Use | 0.04 | 0.02 | -0.01 | 0.08 | 96.86 | 1.56 | 0.123 |  |  |  |

B – unstandardized regression coefficient; SE – standard error of B; 95% CI – 95% confidence interval, df- degrees of freedom, t- test, p- p-value; ICC- intraclass correlation coefficient; σ^2^_participants_- random intercept variance; σ^2^_residual_- residual variance

Supplemental Table 4. Linear mixed effects models for estimating an average relief within different groups of anxiety treatment, with route of administration (ROA; inhaling vs. other) as a predictor variable.

| Groups | N_Sessions_ | N_Participants_ | Predictor | B | SE | 95% CI | | df | t | p | ICC | σ^2^_participants_ | σ^2^_residual_ |
| --- | --- | --- | --- | --- | --- | --- | --- | --- | --- | --- | --- | --- | --- |
| 1. Only MC | 5488 | 326 | Intercept | 3.58 | 0.11 | 3.37 | 3.79 | 431.93 | 33.19 | < .001 | 0.56 | 2.78 | 2.19 |
|  |  |  | ROA (ref. inhaling) | 0 | 0.12 | -0.22 | 0.23 | 5212.36 | 0.02 | 0.985 |  |  |  |
| 2. MC + Drugs | 995 | 158 | Intercept | 3.29 | 0.18 | 2.94 | 3.64 | 191.15 | 18.53 | < .001 | 0.53 | 2.69 | 2.42 |
|  |  |  | ROA (ref. inhaling) | 0.32 | 0.26 | -0.18 | 0.82 | 868.07 | 1.24 | 0.215 |  |  |  |
| 3. MC + Activity | 3040 | 259 | Intercept | 3.56 | 0.13 | 3.3 | 3.83 | 329.15 | 26.73 | < .001 | 0.6 | 3.27 | 2.17 |
|  |  |  | ROA (ref. inhaling) | -0.11 | 0.15 | -0.4 | 0.19 | 2901.93 | -0.71 | 0.477 |  |  |  |
| 4. MC + Drugs + Activity | 1105 | 139 | Intercept | 3.1 | 0.19 | 2.73 | 3.48 | 173.06 | 16.05 | < .001 | 0.64 | 3.72 | 2.08 |
|  |  |  | ROA (ref. inhaling) | -0.15 | 0.2 | -0.55 | 0.24 | 1099.85 | -0.76 | 0.446 |  |  |  |

B – unstandardized regression coefficient; SE – standard error of B; 95% CI – 95% confidence interval, df- degrees of freedom, t- test, p- p-value; ICC- intraclass correlation coefficient; σ^2^_participants_- random intercept variance; σ^2^_residual_- residual variance

Supplemental Table 5. Linear mixed effects models for estimating an average relief within different groups of anxiety treatment, with method of consumption (ROA; Smoking vs. Vape/DAB) as a predictor variable.

| Groups | N_Sessions_ | N_Participants_ | Predictor | B | SE | 95% CI | | | df | t | p | ICC | σ^2^_participants_ | σ^2^_residual_ |
| --- | --- | --- | --- | --- | --- | --- | --- | --- | --- | --- | --- | --- | --- | --- |
| 1. Only MC | 4737 | 302 | Intercept | 3.52 | 0.1 | 3.31 | 3.72 | 313.74 | | 33.8 | < .001 | 0.55 | 2.69 | 2.21 |
|  |  |  | ROA (ref. Smoke) | -0.15 | 0.09 | -0.32 | 0.03 | 4487.47 | | -1.66 | 0.096 |  |  |  |
| 2. MC + Drugs | 844 | 139 | Intercept | 3.49 | 0.18 | 3.14 | 3.84 | 128.76 | | 19.38 | < .001 | 0.54 | 2.88 | 2.43 |
|  |  |  | ROA (ref. Smoke) | -0.01 | 0.25 | -0.5 | 0.48 | 635.61 | | -0.03 | 0.974 |  |  |  |
| 3. MC + Activity | 2538 | 233 | Intercept | 3.48 | 0.13 | 3.23 | 3.74 | 232.81 | | 26.44 | < .001 | 0.61 | 3.26 | 2.08 |
|  |  |  | ROA (ref. Smoke) | 0.08 | 0.14 | -0.19 | 0.34 | 2249.62 | | 0.56 | 0.579 |  |  |  |
| 4. MC + Drugs + Activity | 840 | 119 | Intercept | 3.04 | 0.21 | 2.62 | 3.46 | 153.51 | | 14.35 | < .001 | 0.63 | 3.66 | 2.18 |
|  |  |  | ROA (ref. Smoke) | 0.22 | 0.27 | -0.31 | 0.75 | 762.41 | | 0.82 | 0.413 |  |  |  |

B – unstandardized regression coefficient; SE – standard error of B; 95% CI – 95% confidence interval, df- degrees of freedom, t- test, p- p-value; ICC- intraclass correlation coefficient; σ^2^_participants_- random intercept variance; σ^2^_residual_- residual variance

Supplemental Table 6. Linear mixed effects models for estimating average relief within different groups of anxiety treatment, testing differences between novice and experienced MC patients.

| Groups | N_Sessions_ | N_Participants_ | Predictor | B | SE | 95% CI | | df | t | p | ICC | σ^2^_participants_ | σ^2^_residual_ |
| --- | --- | --- | --- | --- | --- | --- | --- | --- | --- | --- | --- | --- | --- |
| 1. Only MC | 5502 | 328 | Intercept | 3.6 | 0.17 | 3.26 | 3.94 | 342.5 | 20.71 | < .001 | 0.56 | 2.83 | 2.19 |
|  |  |  | Used MC at enrollment (ref. No) | -0.09 | 0.35 | -0.77 | 0.59 | 342.5 | -0.25 | 0.8 |  |  |  |
| 2. MC + Drugs | 1000 | 159 | Intercept | 3.41 | 0.24 | 2.93 | 3.88 | 116.48 | 13.96 | < .001 | 0.53 | 2.71 | 2.44 |
|  |  |  | Used MC at enrollment (ref. No) | -0.01 | 0.49 | -0.97 | 0.94 | 116.48 | -0.03 | 0.977 |  |  |  |
| 3. MC + Activity | 3050 | 260 | Intercept | 3.33 | 0.2 | 2.93 | 3.73 | 258.67 | 16.33 | < .001 | 0.61 | 3.33 | 2.16 |
|  |  |  | Used MC at enrollment (ref. No) | 0.44 | 0.41 | -0.36 | 1.24 | 258.67 | 1.07 | 0.285 |  |  |  |
| 4. MC + Drugs + Activity | 1111 | 141 | Intercept | 2.94 | 0.28 | 2.38 | 3.5 | 154.52 | 10.31 | < .001 | 0.64 | 3.76 | 2.08 |
|  |  |  | Used MC at enrollment (ref. No) | 0.22 | 0.57 | -0.9 | 1.33 | 154.52 | 0.38 | 0.704 |  |  |  |
| 5. Only Drugs | 160 | 63 | Intercept | 0.78 | 0.26 | 0.27 | 1.29 | 38.96 | 3.03 | 0.004 | 0.47 | 1.64 | 1.83 |
|  |  |  | Used MC at enrollment (ref. No) | 0.81 | 0.51 | -0.21 | 1.82 | 38.96 | 1.57 | 0.124 |  |  |  |
| 6. Drugs + Activity | 96 | 38 | Intercept | 0.82 | 0.3 | 0.22 | 1.42 | 24.36 | 2.72 | 0.012 | 0.5 | 1.61 | 1.62 |
|  |  |  | Used MC at enrollment (ref. No) | 0.53 | 0.6 | -0.67 | 1.73 | 24.36 | 0.88 | 0.388 |  |  |  |
| 7. Only Activity | 245 | 83 | Intercept | 1.15 | 0.18 | 0.79 | 1.51 | 58.64 | 6.35 | < .001 | 0.46 | 1.06 | 1.27 |
|  |  |  | Used MC at enrollment (ref. No) | 0.11 | 0.36 | -0.6 | 0.82 | 58.64 | 0.3 | 0.762 |  |  |  |

*b* – unstandardized regression coefficient; SE – standard error of *b*; 95% CI – 95% confidence interval, df- degrees of freedom, t- test, p- p-value; ICC- intraclass correlation coefficient; σ^2^_participants_- random intercept variance; σ^2^_residual_- residual variance

Supplemental Table 7. Linear mixed effects models for estimating average relief within different groups of anxiety treatment among novice MC patients – sensitivity analysis.

|  |  |  | 95% Confidence Intervals | | |  |  |
| --- | --- | --- | --- | --- | --- | --- | --- |
| Effect | Estimate | SE | Lower | Upper | df | t | p |
| (Intercept) | 2.232 | 0.322 | 1.6 | 2.863 | 40.869 | 6.934 | < .001 |
| 2. MC + Drugs - 1. Only MC | -0.07 | 0.198 | -0.459 | 0.319 | 907.394 | -0.354 | 0.723 |
| 3. MC + Activity - 1. Only MC | -0.216 | 0.167 | -0.544 | 0.113 | 920.136 | -1.289 | 0.198 |
| 4. MC + Drugs + Activity - 1. Only MC | -0.379 | 0.259 | -0.887 | 0.128 | 927.027 | -1.466 | 0.143 |
| 5. Only Drugs - 1. Only MC | -2.358 | 0.265 | -2.877 | -1.838 | 912.888 | -8.908 | < .001 |
| 6. Drugs + Activity - 1. Only MC | -1.522 | 0.342 | -2.194 | -0.85 | 918.69 | -4.443 | < .001 |
| 7. Only Activity - 1. Only MC | -2.202 | 0.26 | -2.712 | -1.691 | 913.871 | -8.461 | < .001 |
| ICC | 0.602 |  |  |  |  |  |  |
| σ^2^_participants_ | 3.535 |  |  |  |  |  |  |
| σ^2^_residual_ | 2.338 |  |  |  |  |  |  |

Supplemental Table 8. Results of pairwise comparisons of average relief between different anxiety treatment groups among novice MC users.

| Group Comparison |  | d | SE | t | df | p_bonferroni_ |
| --- | --- | --- | --- | --- | --- | --- |
| 1. Only MC | 2. MC + Drugs | 0.07 | 0.198 | 0.354 | 907.394 | 1 |
| 1. Only MC | 3. MC + Activity | 0.216 | 0.167 | 1.289 | 920.136 | 1 |
| 1. Only MC | 4. MC + Drugs + Activity | 0.379 | 0.259 | 1.466 | 927.027 | 1 |
| 1. Only MC | 5. Only Drugs | 2.358 | 0.265 | 8.908 | 912.888 | < .001 |
| 1. Only MC | 6. Drugs + Activity | 1.522 | 0.342 | 4.443 | 918.69 | < .001 |
| 1. Only MC | 7. Only activity | 2.202 | 0.26 | 8.461 | 913.871 | < .001 |
| 2. MC + Drugs | 3. MC + Activity | 0.146 | 0.225 | 0.646 | 913.385 | 1 |
| 2. MC + Drugs | 4. MC + Drugs + Activity | 0.309 | 0.29 | 1.064 | 921.188 | 1 |
| 2. MC + Drugs | 5. Only Drugs | 2.287 | 0.278 | 8.219 | 902.847 | < .001 |
| 2. MC + Drugs | 6. Drugs + Activity | 1.451 | 0.356 | 4.083 | 912.742 | 0.001 |
| 2. MC + Drugs | 7. Only activity | 2.131 | 0.3 | 7.113 | 910.104 | < .001 |
| 3. MC + Activity | 4. MC + Drugs + Activity | 0.163 | 0.262 | 0.624 | 923.385 | 1 |
| 3. MC + Activity | 5. Only Drugs | 2.142 | 0.275 | 7.801 | 911.915 | < .001 |
| 3. MC + Activity | 6. Drugs + Activity | 1.306 | 0.349 | 3.745 | 917.825 | 0.004 |
| 3. MC + Activity | 7. Only activity | 1.986 | 0.259 | 7.66 | 909.616 | < .001 |
| 4. MC + Drugs + Activity | 5. Only Drugs | 1.979 | 0.326 | 6.061 | 917.66 | < .001 |
| 4. MC + Drugs + Activity | 6. Drugs + Activity | 1.143 | 0.37 | 3.084 | 915.965 | 0.044 |
| 4. MC + Drugs + Activity | 7. Only activity | 1.823 | 0.331 | 5.502 | 920.316 | < .001 |
| 5. Only Drugs | 6. Drugs + Activity | -0.836 | 0.374 | -2.237 | 908.518 | 0.536 |
| 5. Only Drugs | 7. Only activity | -0.156 | 0.32 | -0.487 | 902.661 | 1 |
| 6. Drugs + Activity | 7. Only activity | 0.68 | 0.388 | 1.754 | 913.105 | 1 |

Supplementary Table 9. Linear mixed effects model for estimating difference in anxiety relief between novice and experienced MC patients across the 45 days of study.

|  |  |  | 95% Confidence Intervals | | |  |  |
| --- | --- | --- | --- | --- | --- | --- | --- |
| Effect | Estimate | SE | Lower | Upper | df | t | p |
| (Intercept) | 3.61 | 0.17 | 3.27 | 3.96 | 343.08 | 20.77 | < .001 |
| MC at the enrollment (ref. Yes) | -0.15 | 0.35 | -0.83 | 0.53 | 343.08 | -0.43 | 0.67 |
| Day | 0.00 | 0.00 | 0.00 | 0.01 | 5438.12 | 1.26 | 0.21 |
| MC use at enrollment X Day | -0.02 | 0.01 | -0.03 | 0.00 | 5438.12 | -2.49 | 0.01 |
| ICC | 0.56 |  |  |  |  |  |  |
| σ^2^_participants_ | 2.83 |  |  |  |  |  |  |
| σ^2^_residual_ | 2.19 |  |  |  |  |  |  |
